# Expression Pattern of the Aspartyl-tRNA Synthetase DARS in the Human Brain

**DOI:** 10.3389/fnmol.2018.00081

**Published:** 2018-03-20

**Authors:** Dominik Fröhlich, Alexandra K. Suchowerska, Carola Voss, Ruojie He, Ernst Wolvetang, Georg von Jonquieres, Cas Simons, Thomas Fath, Gary D. Housley, Matthias Klugmann

**Affiliations:** ^1^Translational Neuroscience Facility and Department of Physiology, School of Medical Sciences, University of New South Wales, Sydney, NSW, Australia; ^2^Neurodegenerative and Repair Unit, School of Medical Sciences, University of New South Wales, Sydney, NSW, Australia; ^3^Stem Cell Engineering Group, Australian Institute for Bioengineering and Nanotechnology, The University of Queensland, Brisbane, QLD, Australia; ^4^Department of Neurology, First Affiliated Hospital, Sun Yat-sen University, Guangzhou, China; ^5^Institute for Molecular Bioscience, The University of Queensland, Brisbane, QLD, Australia

**Keywords:** DARS, HBSL, leukodystrophy, myelin, aminoacyl-tRNA synthetase, aspartyl-tRNA synthetase, translation

## Abstract

Translation of mRNA into protein is an evolutionarily conserved, fundamental process of life. A prerequisite for translation is the accurate charging of tRNAs with their cognate amino acids, a reaction catalyzed by specific aminoacyl-tRNA synthetases. One of these enzymes is the aspartyl-tRNA synthetase DARS, which pairs aspartate with its corresponding tRNA. Missense mutations of the gene encoding DARS result in the leukodystrophy hypomyelination with brainstem and spinal cord involvement and leg spasticity (HBSL) with a distinct pattern of hypomyelination, motor abnormalities, and cognitive impairment. A thorough understanding of the DARS expression domains in the central nervous system is essential for the development of targeted therapies to treat HBSL. Here, we analyzed endogenous DARS expression on the mRNA and protein level in different brain regions and cell types of human post mortem brain tissue as well as in human stem cell derived neurons, oligodendrocytes, and astrocytes. DARS expression is significantly enriched in the cerebellum, a region affected in HBSL patients and important for motor control. Although obligatorily expressed in all cells, DARS shows a distinct expression pattern with enrichment in neurons but only low abundance in oligodendrocytes, astrocytes, and microglia. Our results reveal little homogeneity across the different cell types, largely matching previously published data in the murine brain. This human gene expression study will significantly contribute to the understanding of DARS gene function and HBSL pathology and will be instrumental for future development of animal models and targeted therapies. In particular, we anticipate high benefit from a gene replacement approach in neurons of HBSL mouse models, given the abundant endogenous DARS expression in this lineage cell.

## Introduction

Translation of mRNA into protein is a key mechanism of life that is evolutionarily conserved across all cells and organisms. Before translation is initiated each amino acid has to be linked to its cognate tRNA, a two-step reaction also known as tRNA charging (Antonellis and Green, 2008). This reaction is catalyzed by a group of enzymes named aminoacyl-tRNA synthetases (ARSs). Of the 36 human ARSs, 16 are located in the cytoplasm, 17 in mitochondria, and three can act in both compartments (Antonellis and Green, 2008). In order to link all 20 amino acids with their corresponding tRNAs, the charging of glutamate and proline in the cytoplasm is catalyzed by one common ARS, the glutamyl-prolyl-tRNA synthetase (Berthonneau and Mirande, 2000). All cytoplasmic ARSs are denoted by their designated single-letter amino acid abbreviation followed by the acronym ARS (e.g., AARS for alanyl-tRNA-synthetase). Mitochondrial ARSs are named the same way followed by the number 2 (e.g., AARS2 for the mitochondrial alanyl-tRNA-synthetase). In humans and other mammals, nine of the 19 cytosolic ARSs together with three ARS-interacting multifunctional proteins (AIMP1, 2, and 3) are organized in the so called multi synthetase complex (MSC) (Yao and Fox, 2013). Apart from their canonical function in translation, multiple non-canonical functions have been assigned to individual ARSs. These secondary functions include homeostasis of amino acid metabolism, mediation of inflammatory and immune responses, angiogenesis signaling, and control of stress responses and apoptosis (for review, see Guo and Schimmel, 2013).

There is no redundancy among ARS enzymes and their importance becomes apparent when they are genetically mutated. Interestingly, mutations in cytoplasmic as well as the mitochondrial ARSs primarily manifest in deficits of the central and peripheral nervous system, indicating that neural cells are particularly susceptible to disturbances in protein translation. The spectrum of neurological diseases comprises encephalopathies, Charcot-Marie-Tooth neuropathies, cerebellar ataxia, peripheral neuropathies, and leukodystrophies (Antonellis and Green, 2008).

Homozygous as well as compound heterozygous missense mutations of the gene encoding the cytoplasmic aspartyl-tRNA synthetase DARS, which is responsible for the charging of aspartate, cause the severe leukodystrophy hypomyelination with brainstem and spinal cord involvement and leg spasticity (HBSL) (Taft et al., 2013). Most HBSL cases identified to date show an infantile onset with severe disease progression. Neuroanatomical abnormalities include hypomyelination of the supratentorial white matter, cerebellar peduncles, anterior brainstem, and dorsal columns of the spinal cord. Neurologically, HBSL patients present with motor paralysis, progressive leg spasticity, cerebellar dysfunction, nystagmus, variable cognitive impairment, and epilepsy (Taft et al., 2013; Wolf et al., 2015). Recently, two cases with late adolescence onset, a milder disease course, and symptoms similar to a steroid responsive neuroinflammatory disorder have been described, which broadens the clinical spectrum of HBSL and suggests that the number of undiagnosed cases might be higher than originally anticipated (Wolf et al., 2015). HBSL could share a common pathomechanism with leukoencephalopathy with brainstem and spinal cord involvement and elevated lactate (LBSL), caused by missense mutations of the mitochondrial *DARS2* gene, as patients afflicted by these conditions display matching symptoms (Scheper et al., 2007). A lack of enzymatic activity of either protein, however, cannot be compensated by action of the other, as *Dars*-null (Fröhlich et al., 2016) as well as *Dars2*-null mice (Dogan et al., 2014) die before birth.

In a previous study, we characterized DARS expression domains in mice and found surprisingly little homogeneity across brain regions and neural cell types (Fröhlich et al., 2016). In mice, DARS expression is enriched in neuronal subpopulations of the cerebellum, hippocampus, and cortex with only low abundance immunoreactivity in macroglia.

The aim of this study was to extend our knowledge of DARS expression domains to the human brain, a prerequisite for the creation of relevant disease models and therapeutic strategies. Therefore, we comprehensively assessed DARS mRNA and protein levels in non-diseased human brain tissue. Broadly, we found that RNA amounts mirrored DARS protein. In line with the results observed in mice, the highest DARS levels were present in the cerebellum. Analysis of DARS protein expression at a cell population level revealed DARS immunoreactivity in all major neural cell types with strong enrichment in neuronal subpopulations. This clear overlap of expression domains in the brain of mice and humans will increase the translational relevance of mouse studies and can facilitate the development of targeted therapeutic interventions in the future.

## Materials and Methods

### Ethics Statement

All procedures were approved by the UNSW Sydney Human Research Ethics Advisory Panel D and the University of Queensland Human Research Ethics Committee.

### Brain Tissue

Frozen tissue blocks (immunoblotting and qRT-PCR) or paraformaldehyde-fixed, paraffin-embedded sections (4 μm; immunohistochemistry) from six different brain regions [motor cortex, hippocampus, cerebellum, brainstem (pons), striatum, and corpus callosum] were obtained from the New South Wales Brain Bank (NSWBB; project no. PID391). Tissue from all six brain regions was taken from five male subjects aged 55–57 years. These patients died due to cardiovascular related complications and did not have any overt brain pathology. The post-mortem interval ranged from 12 to 39 h. Immunoblotting and qRT-PCR was conducted on tissue from all five subjects. Immunohistochemistry was undertaken on tissue from three of the five individuals.

### Western-Blotting

HEK 293 cells were either transfected with a control plasmid (pAM/CBA-EGFP) or with a tagged DARS plasmid (pAM/CBA-Flag-HA-DARS) using standard calcium phosphate transfection. After 3 days, cells were lysed in lysis buffer (50 mM Tris-Cl, pH 7.4, 1 mM EDTA pH 8.0, 250 mM NaCl, and 1% Triton-X) containing protease inhibitors (Complete, Roche).

Sample preparation of the brain tissue was performed as described previously (Fröhlich et al., 2016). Briefly, the tissue was homogenized in liquid nitrogen using mortar and pestle and subsequently sonicated in lysis buffer (50 mM Tris-Cl, pH 7.4, 1 mM EDTA pH 8.0, 250 mM NaCl, and 1% Triton-X) containing protease inhibitors (Complete, Roche).

Protein concentration of the lysates was measured using the Bradford protein assay (Bio-Rad no. 5000006). 10 μg of protein from the HEK 293 cell lysate or 30 μg of protein from each brain region was mixed with 5x sample buffer (15 g SDS, 15.6 ml 2 M Tris pH 6.8, 57.5 g glycerol, 16.6 ml β-mercaptoethanol), loaded onto a 10% acrylamide gel, separated by SDS-PAGE, and transferred onto PVDF membranes (Bio-Rad no. 162-0177). After blocking with 4% milk containing 0.1% Tween, the membrane was probed with the following primary antibodies at 4°C over night: mαDARS (1:500; SantaCruz no. sc-393275; raised against amino acids 170–467 of human DARS), rbαDARS (1:500; Novus Biological no. NBP1-85937; raised against amino acids 1–135 of human DARS), rbαHA (Cell Signaling no. 3724), mαHA (Cell Signaling no. 2367), rbαGAPDH (1:5000, Cell Signaling no. 2118S), and mαβ-Actin clone C4 (1:10000, Sigma-Aldrich no. mab1501). After washing 3 times with 0.1% Tween in PBS, HRP-conjugated secondary antibodies (Dianova, Hamburg, Germany) in 4% milk containing 0.1% Tween were applied for 2 h at room temperature. Membranes were developed using Clarity western ECL substrate (Bio-Rad no. 170-5060) and imaged using the GelDoc system (Bio-Rad). DARS protein levels in different brain regions were normalized to the housekeeping proteins β-Actin or GAPDH and displayed in relation to the cortex.

### RNA Isolation and qRT-PCR

Sample preparation and qRT-PCR was performed as described previously (Fröhlich et al., 2016). The tissue was homogenized in liquid nitrogen using mortar and pestle and RNA was extracted using the RNeasy MiniKit according to the manufacturer’s instructions (Qiagen no. 74106) including on-column DNase digestion (RNase-Free DNase Set, Qiagen no. 79254). Following cDNA synthesis using the High Capacity cDNA Reverse Transcription Kit (Applied Biosystems no. 4368813), qRT-PCR was performed in a StepOnePlus Real-Time PCR system (Applied Biosystems) utilizing TaqMan assays (Applied Biosystems) for *DARS* (Hs00154683_m1), *DARS2* (Hs00216620_m1), and *β-Actin* (Hs01060665_g1). The comparative CT method (ΔΔCT) for relative quantification of expression was used and normalized to the housekeeping gene *β-Actin*.

### Immunohistochemistry

Formalin-fixed and paraffin-embedded sections (4 μm) from the motor cortex, hippocampus, cerebellum, striatum, and corpus callosum were cut in the coronal plane; sections from the brainstem (pons) were cut in the transverse plane. After rehydration and antigen retrieval (10 mM citrate buffer, 120°C for 1 min), sections were permeabilized in 0.5% TritonX-100 for 3 min and then blocked with 10% normal goat serum in phosphate buffered saline (PBS). Slides were incubated overnight at 4°C with the following primary antibodies: mαDARS (1:50; SantaCruz no. sc-393275), rbαNeuN (1:40; Cell Signaling no. 12943S), rbαASPA (1:300; Mersmann et al., 2011), rbαGFAP (1:300; DAKO no. Z0334), and rbαIba1 (1:300; Wako no. 019-19741). Slides were then washed in PBS and incubated with goat secondary antibodies (αmouse-Alexa555, αrabbit-Alexa488; 1:300; Thermo Fisher) for 1 h at room temperature. Slides were washed with PBS, incubated for 5 min with DAPI, and washed again with PBS. Afterward, slides were washed in 70% ethanol before applying autofluorescence eliminator reagent (Millipore no. 2160). Slides were washed again in 70% ethanol, then washed in PBS before mounting in ProLong Gold antifade reagent (Thermo Fisher no. P10144). Images were taken with a Zeiss Z1 AxioExaminer NLO710 confocal microscope and image processing was performed using ImageJ software.

Immunoperoxidase labeling using diaminobenzidine (DAB) substrate was performed on sections incubated with biotin conjugated gtαmouse secondary antibody (1:250; Jackson ImmunoResearch no. 111-065-003). Subsequently, sections were stained using the DAB substrate kit for peroxidase (Vector Laboratories no. SK-4100) according to the manual. Stained sections were digitized with an Aperio slide scanner (Leica) and image processing was performed using the Aperio ImageScope (Leica) software.

### hESC Derived Cortical Neurons

Cortical neurons were differentiated from human embryonic stem cells (hESCs; H9) according to the protocol by Shi et al. (2012). Pluripotent stem cell colonies at 80% confluence were used as the starting material. After a 10-day neural induction with 10 μM SB431542 (Tocris Bioscience no. 1614) and 1 μM Dorsomorphin (Sigma no. P5499), neuroepithelial cells were passaged and cultured in neural maintenance medium, according to the protocol, on ECM (1:100, Sigma no. E1270) coated plates for 14–18 additional days. After a first passage by cutting and replating the neural rosettes, neural stem cells (NSCs) were expanded in maintenance medium and passaged with Accutase (StemPro, Life Technologies no. A1110501). To promote terminal differentiation, NSCs were plated on poly-L-ornithine/laminin-coated plates (0.1 mg/ml poly-L-ornithine hydrobromide, 30,000–70,000 kDa, Sigma no. P3655, and 5 μg/ml mouse laminin, Invitrogen no. 23017-015) and cultured in neural maintenance medium in the presence of 20 ng/ml human BDNF (Peprotech no. 450-02), 20 ng/ml human GDNF (Peprotech no. 450-10), 200 nM ascorbic acid (Sigma no. A4544), 0.5 mM dibutyryl cAMP sodium salt (Sigma no. D0627) and 1 μg/ml laminin.

### Human iPSC Derived Oligodendrocytes

Oligodendrocytes were differentiated from human induced pluripotent stem cells (iPSCs; C32) following the protocol of Douvaras and Fossati (2015). Briefly, iPSCs were seeded at 200,000 cells per six well as single cells on ECM coated plates. After an 8-day neural induction period with 10 μM SB431542, 250 nM Stemolecule^TM^ LDN -193189 (Bioscientific no. 04-0074) and 100 nM retinoic acid (Sigma no. R2625), medium was switched to N2 medium until day 12. On day 12, cells were mechanically disassociated and consecutively cultured as cell aggregates in suspension in N2B27 medium in ultra-low attachment plates (Sigma no. CLS3471) until day 20. Afterward, medium was switched to PDGF medium. After 10 additional days (total 30 days) in suspension, 20 cell aggregates per six well were plated on poly-L-ornithine/laminin-coated plates (0.05 mg/ml poly-L-ornithine hydrobromide, 30,000–70,000 kDa, 20 μg/ml mouse laminin) up to day 75. Detailed medium compositions and reagent companies were followed precisely as published.

### Primary Human Astrocytes

Primary human Astrocytes were obtained from ScienCell (1800, P2) and cultured as per manufacturer’s description in astrocyte medium (AM, ScienCell no. 1801).

### Immunocytochemistry

Cells were fixed in 4% paraformaldehyde (ProSciTech no. C004) for 15 min at room temperature and washed twice in PBS. After quenching the samples in 50 mM NH_4_Cl (Sigma no. 254134) for 10 min, neurons were permeabilized in 0.3% Triton X-100 (Sigma no. T8787) in PBS for 15 min, and subsequently blocked in blocking buffer (0.2% bovine serum albumin (Sigma no. 05470), 0.2% fish skin gelatine, and 0.1% Triton X-100 in PBS). Cells were incubated overnight at 4°C with the following primary antibodies diluted in blocking buffer: rbαDARS (1:100; LSBio company no. LS-C167270), chαβIIITubulin (1:500; Merck Millipore no. AB9354), mαGFAP (1:250; Merck Millipore no. MAB360), mαO4 (1:100; Merck Millipore no. MAB345). Afterward, samples were rinsed twice with PBS for 5 min and stained with the following secondary antibodies in blocking buffer for 2 h at room temperature: gtαrabbit-Alexa488 (1:1000; Invitrogen no. R37116), dkαchicken-Alexa647 (1:1000; Merck Millipore no. AP194SA6), gtαmouse-Alexa488 (1:500; Invitrogen no. A-21043), gtαrabbit-Alexa633 (1:1000; Invitrogen no. A-21070). Cells were rinsed in PBS before mounting them in mounting medium (Glycergel, DAKO no. C056330-2). Images were taken with a Zeiss 710 confocal laser scanning microscope.

### Statistics

Graphs and statistical analyses were performed with the GraphPad Prism 7 software. Multiple comparisons one-way ANOVA (Fisher’s LSD test) was used for statistical analysis, subsequent to validation of normal distribution of data.

## Results

### Region Specific DARS mRNA and Protein Expression

**FIGURE 1:**
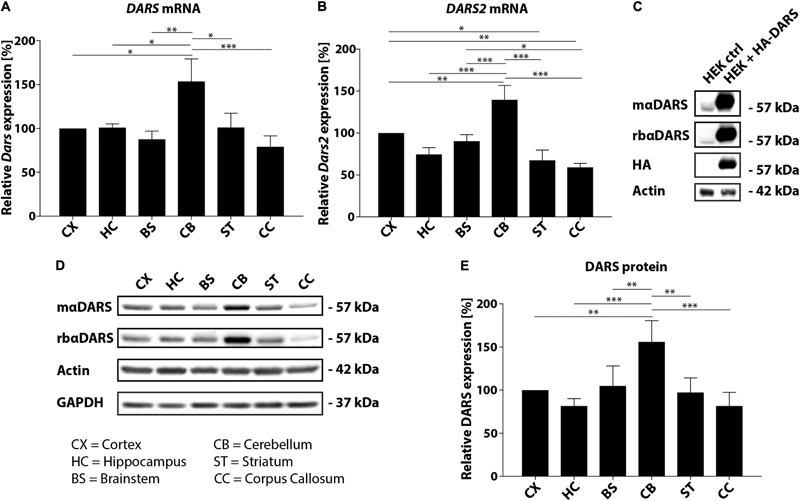
DARS expression in the adult human brain. **(A,B)** Levels of *DARS* **(A)** and *DARS2* **(B)** mRNA across different brain regions [CX, motor cortex; HC, hippocampus; BS, brainstem (pons); CB, cerebellum; ST, striatum; CC, corpus callosum] determined by qRT-PCR. *DARS* and *DARS2* mRNA were normalized to the housekeeping gene *β-Actin* (*n* = 5). **(C)** Western-blot detection of DARS in lysates from HEK 293 cells ectopically expressing human DARS including an N-terminal FLAG-HA-tag or a control plasmid. DARS protein was detected with a monoclonal mouse antibody from SantaCruz (mαDARS), or a polyclonal rabbit antibody from Novus Biologicals (rbαDARS), or an HA antibody. β-Actin served as internal standard. **(D)** Representative Western-blot depicting the expression of DARS, β-Actin, and GAPDH across different brain regions. **(E)** Levels of DARS protein (detected with mαDARS) normalized to β-Actin (*n* = 5). Data represent mean ± SEM (^∗^*p* < 0.05, ^∗∗^*p* < 0.01, ^∗∗∗^*p* < 0.001; one-way ANOVA).

In this study, we analyzed the expression of *DARS* mRNA and DARS protein in human post-mortem brain tissue. Characterization of endogenous *DARS* mRNA levels across different brain regions using qRT-PCR revealed significantly higher expression in the cerebellum compared to other areas analyzed including motor cortex, hippocampus, brainstem (pons), striatum, and corpus callosum (**Figure 1A**). Interestingly, the mitochondrial homolog *DARS2* showed a similar distribution with the highest mRNA levels also in the cerebellum (**Figure 1B**). In order to compare DARS protein levels across brain regions, we first characterized two different DARS antibodies to determine their specificity for the 501 amino acid human DARS protein. The first antibody was a monoclonal mouse antibody from SantaCruz (mαDARS), which was raised against amino acids 170–467 of human DARS protein. The second antibody was a polyclonal rabbit antibody from Novus Biologicals (rbαDARS), which was developed against recombinant protein corresponding to amino acids 1–135 of human DARS. HEK-293 cells were either transfected with a control plasmid or with a DARS plasmid containing an N-terminal FLAG-HA-tag, lysed after 3 days, and analyzed using Western-blotting. Both antibodies detected endogenous DARS as well as the slightly larger tagged variant (**Figure 1C**). Detection with an HA-tag specific antibody revealed only the ectopically expressed version of the protein. Comparison of DARS protein levels across different brain regions using Western-blotting with either the monoclonal mouse αDARS or the polyclonal rabbit αDARS antibody, and normalization to two separate housekeeping proteins, β-Actin or GAPDH, confirmed the mRNA results on a protein level (**Figures 1D,E**). In line with the mRNA levels, DARS protein expression in the cerebellum was significantly increased by 50–100% compared to other brain regions (**Figure 1E**).

### DARS Expression Patterns

**FIGURE 2:**
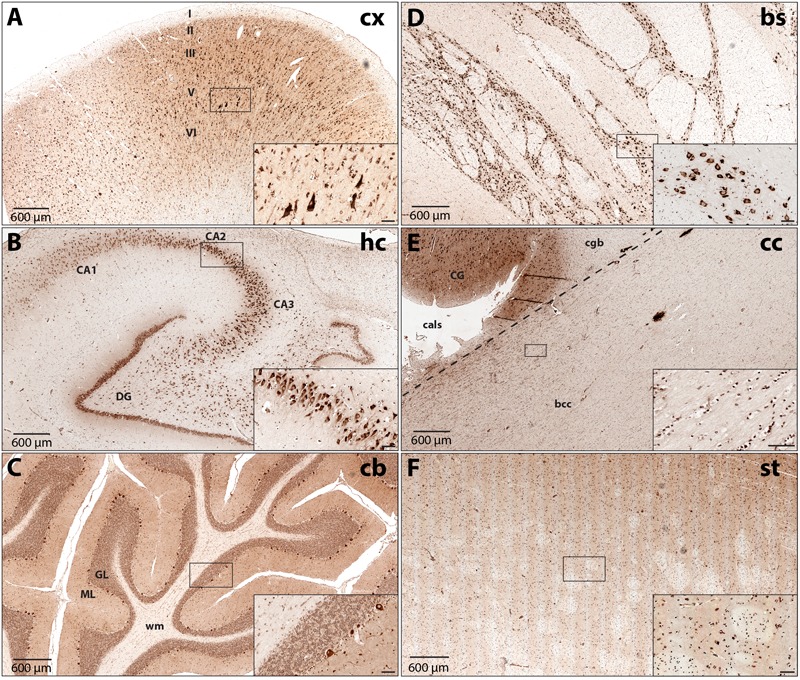
DARS protein expression across different regions of the human brain. Immunohistochemistry of DARS protein in coronal (cx, hc, cb, cc, and st) or transversal (bs) brain sections. DARS is stained with diaminobenzidine (DAB) in brown. Close-ups show specific cells expressing DARS (scale bar: 60 μm). **(A)** In the motor cortex (cx) the different cortical layers are labeled (I–VI). **(B)** In the hippocampus (hc) the fields CA1, CA2, CA3, as well as the dentate gyrus (DG) are depicted. **(C)** In the cerebellum (cb) the molecular (ML) and granular (GL) layer and white matter (wm) are labeled. **(D)** DARS immunoreactivity in the brainstem (bs). **(E)** Labels in the corpus callosum (cc) include bcc (body of corpus callosum), CG (cingulate gyrus), cgb (cingulum bundle), and cals (callosal sulcus). **(F)** DARS immunoreactivity in the striatum (st).

To increase our understanding of sub-region and cell type-specific DARS expression we stained formalin-fixed and paraffin-embedded sections from the same individuals mentioned above using the monoclonal mouse αDARS antibody followed by immunoperoxidase labeling using DAB (**Figure 2** and **Table 1**). The motor cortex, hippocampus, cerebellum, striatum, and corpus callosum were cut in the coronal plane whereas the brainstem (pons) was cut in the transverse plane. In the motor cortex the strongest DARS expression was present in layers II–VI where the cell bodies of pyramidal neurons are located (**Figure 2A**). In particular Betz cells, the large pyramidal cells of layer V, showed strong DARS immunoreactivity in their soma (**Figure 2A**, inset). In the hippocampus, the pyramidal cells of the CA1, CA2, and CA3 region, as well as the granule cells of the dentate gyrus (DG) were strongly DARS positive (**Figure 2B**). The highest DARS expression levels in the cerebellum were present in the large cell bodies of the Purkinje cells as well as in smaller cells also located in the Purkinje cell layer, presumably Bergmann glia (**Figure 2C**). Lower DARS levels were evident in the densely packed granule cells of the granular layer (GL), as well as the more sparsely distributed cells of the molecular layer (ML). In the white matter (wm) of the cerebellum limited DARS immunoreactivity was present. Similarly, in the brainstem strong DARS expression was detected in large cells of the gray matter, whereas the white matter showed only little DARS immunoreactivity (**Figure 2D**). In the corpus callosum only low expression levels were present relative to the neighboring cingulate gyrus (CG), however, the cells that were DARS positive had the appearance and chain-like arrangement of oligodendrocytes (**Figure 2E**). Finally, DARS expressing cells in the striatum were located in the gray matter as well as in the white matter tracts (**Figure 2F**).

**Table 1:** DARS immunoreactivity in different brain regions.

Brain region	DARS expression
**Motor cortex**	
Layer I	+
Layer II	++
Layer III	+++
Layer V	+++
Layer VI	++
**Hippocampus**	
CA1	+++
CA2	++++
CA3	++++
Dentate gyrus	++++
**Cerebellum**	
Molecular layer	++
Granular layer	+++
Purkinje cell layer	++++
White matter	+
**Brainstem**	
Gray matter	++++
White matter	+
**Corpus callosum**	
Body of corpus callosum	+
**Striatum**	
Gray matter	++
White matter	+

*++++, intense immunoreactivity (IR); +++, strong IR; ++, moderate IR; +, weak IR.*

### Cell Type-Specific DARS Expression

We then characterized the cell type-specific DARS expression in coronal sections of the human motor cortex, hippocampus, cerebellum, striatum, and corpus callosum, as well as transversal sections of the brainstem (pons) by immunofluorescence. Cells were co-labeled with the monoclonal mouse αDARS antibody as well as the cell type-specific markers NeuN (neurons), ASPA (oligodendrocytes), GFAP (astrocytes), and IBA1 (microglia).

### DARS Expression in the Cortex

**FIGURE 3:**
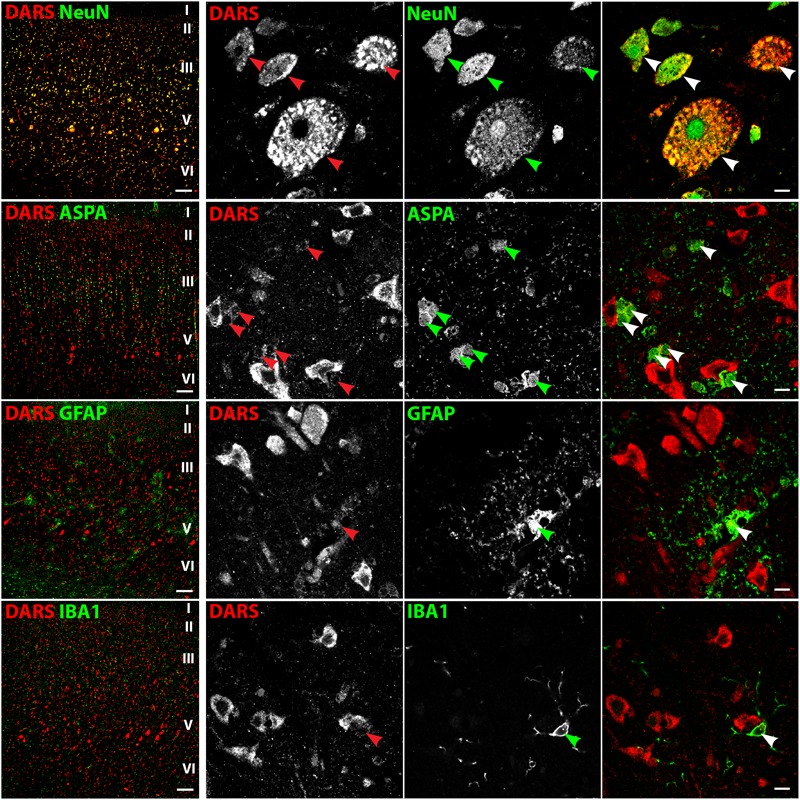
Cell type-specific DARS expression in the motor cortex. Immunofluorescence of DARS (red) and the cell type-specific markers (green) NeuN (neurons), ASPA (oligodendrocytes), GFAP (astrocytes), and IBA1 (microglia). Left panel shows low power magnification of the cortical layers I to VI (scale bar: 200 μm). Right panel displays high power images including separate channels to illustrate cell type-specific as well as subcellular DARS localization (scale bar: 10 μm). Arrowheads highlight cells positive for DARS and the specific cell type marker.

In line with the DAB labeling (**Figure 2A**), the cells with the strongest DARS expression in the motor cortex were located in cortical layers II–VI (**Figure 3**, left column) and were also positive for the neuronal marker NeuN. High power images of individual emission channels as well as merged images (**Figure 3**, right three columns) showed that DARS was also expressed by oligodendrocytes, astrocytes, and microglia, although to a much lower extent than in cortical neurons. In addition, the higher magnification revealed the subcellular localization of DARS. As expected for a cytoplasmic ARS, DARS was mainly located in perinuclear regions of the cell soma.

### DARS Expression in the Hippocampus

**FIGURE 4:**
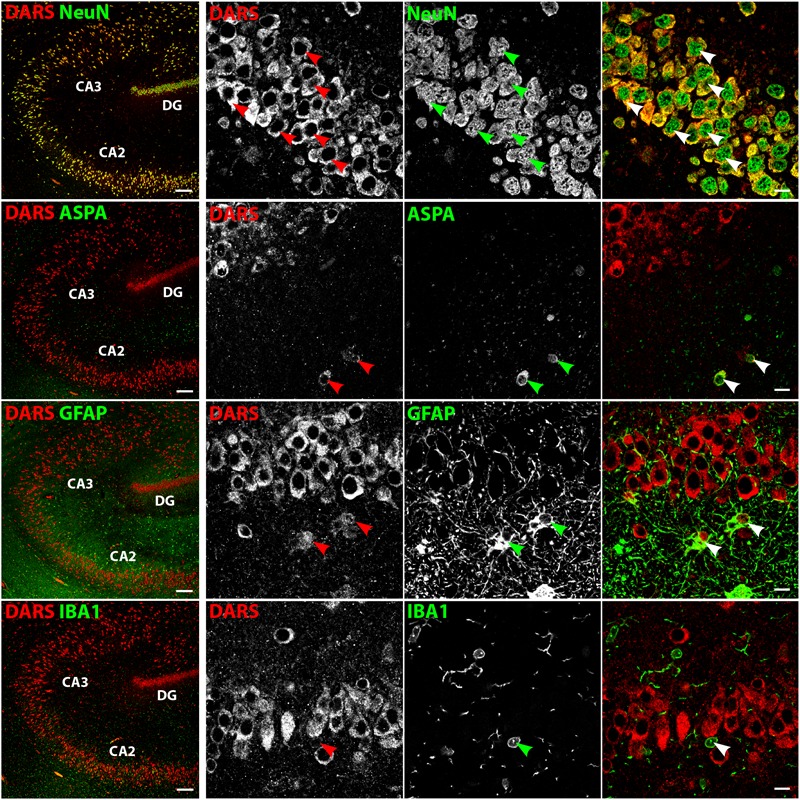
Cell type-specific DARS expression in the hippocampus. Immunofluorescence of DARS (red) and the cell type-specific markers (green) NeuN (neurons), ASPA (oligodendrocytes), GFAP (astrocytes), and IBA1 (microglia). Left panel shows low power magnification of CA2, CA3, and DG (scale bar: 200 μm). Right panel displays high power images including separate channels to illustrate cell type-specific as well as subcellular DARS localization (scale bar: 10 μm). Arrowheads highlight cells positive for DARS and the specific cell type marker.

In the hippocampus the highest DARS expression was observed in cells of the CA1, CA2, and CA3 region, as well as in the DG (**Figure 2B**). These cells were all positive for the neuronal marker NeuN (**Figure 4**, top row). The CA1, CA2, and CA3 regions mainly contain pyramidal cells, whereas the DG consists of granule cells. Again, ASPA positive oligodendrocytes, GFAP positive astrocytes, and IBA1 positive microglia showed DARS expression, which was considerably weaker than in neurons. Especially in microglia, the DARS signal was close to the detection threshold (**Figure 4**, bottom row).

### DARS Expression in the Cerebellum

**FIGURE 5:**
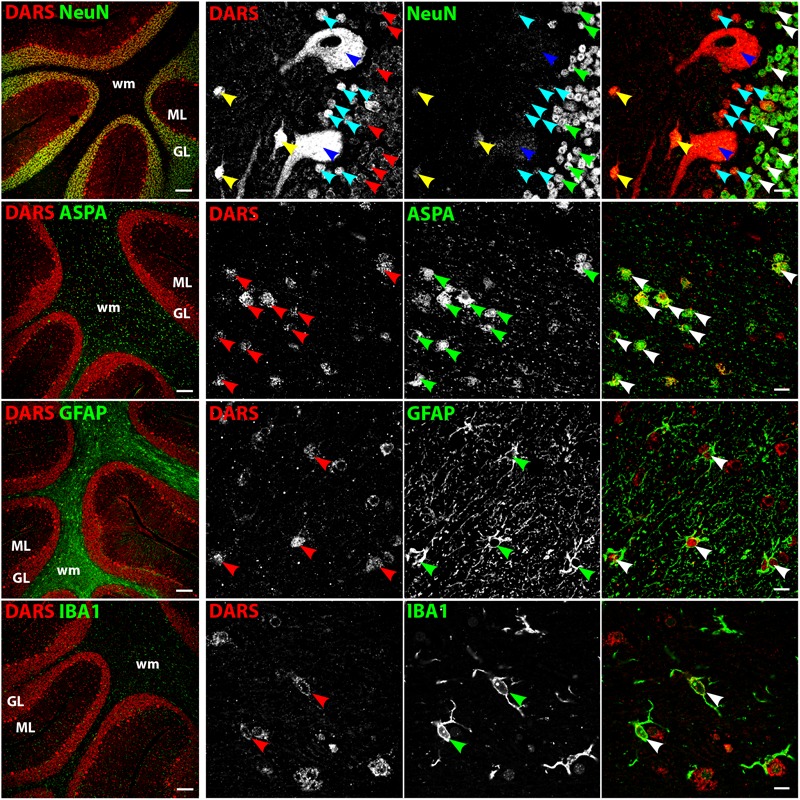
Cell type-specific DARS expression in the cerebellum. Immunofluorescence of DARS (red) and the cell type-specific markers (green) NeuN (neurons), ASPA (oligodendrocytes), GFAP (astrocytes), and IBA1 (microglia). Left panel shows low power magnification of the granular layer (GL), molecular layer (ML), and white matter (wm) (scale bar: 200 μm). Right panel displays high power images including separate channels to illustrate cell type-specific as well as subcellular DARS localization (scale bar: 10 μm). Red, green, and white arrowheads mark cells positive for DARS and the cell type marker. Blue arrowheads highlight Purkinje cells, turquoise arrowheads Bergmann glia, and yellow arrowheads basket cells.

The strongest DARS expression in the cerebellum was present in the Purkinje cell layer, followed by the GL, the more sparsely packed ML, and the white matter, which showed only little DARS immunoreactivity (**Figure 5**). In the Purkinje cell layer the strongest signal was detected in the large cell bodies of Purkinje neurons (**Figure 5**, top row, blue arrowheads). Adjacent to the Purkinje cells were a number of smaller, strongly DARS positive cells. According to their position and size these cells are likely to be Bergmann glia (**Figure 5**, top row, turquoise arrowheads). Next to the Purkinje cells, located in the ML, was another class of strongly DARS expressing cells. Judged by their morphology and position these cells are presumably basket cells (**Figure 5**, top panel, yellow arrowheads). The small granule cells showed only weak DARS expression in their thin cytoplasm (**Figure 5**, top row, red, green, and white arrowheads). Oligodendrocytes (**Figure 5**, second row), astrocytes (**Figure 5**, third row), and microglia (**Figure 5**, bottom row) of the cerebellum were DARS positive but their expression levels were lower compared to neurons.

### DARS Expression in the Brainstem

**FIGURE 6:**
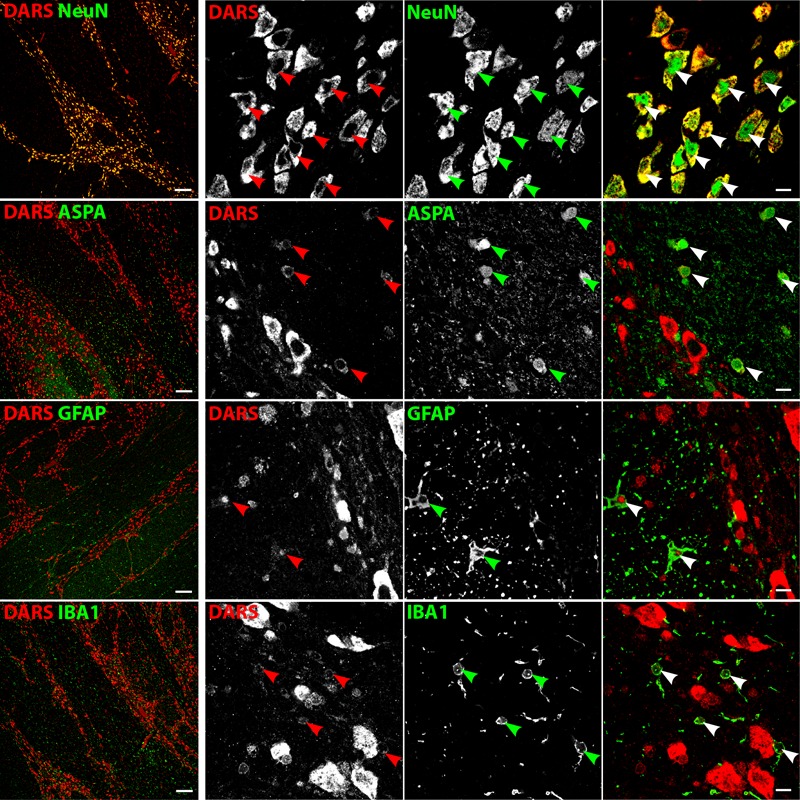
Cell type-specific DARS expression in the brainstem (pons). Immunofluorescence of DARS (red) and the cell type-specific markers (green) NeuN (neurons), ASPA (oligodendrocytes), GFAP (astrocytes), and IBA1 (microglia). Left panel shows low power magnification of the gray and white matter of the pons (scale bar: 200 μm). Right panel displays high power images including separate channels to illustrate cell type-specific as well as subcellular DARS localization (scale bar: 10 μm). Arrowheads indicate DARS-positive cells.

Low power images of the brainstem (**Figure 6**, left column) indicate that DARS is mainly expressed in neurons of the gray matter whereas the DARS signal in the white matter was relatively sparse. Higher magnification images (**Figure 6**, right three columns) showed strong DARS expression in the cytoplasm of neurons (**Figure 6**, top row). In accordance to the other brain regions, the DARS expression level in oligodendrocytes (**Figure 6**, second row), astrocytes (**Figure 6**, third row), and microglia (**Figure 6**, bottom row) was substantially lower than in neurons.

### DARS Expression in the Corpus Callosum

**FIGURE 7:**
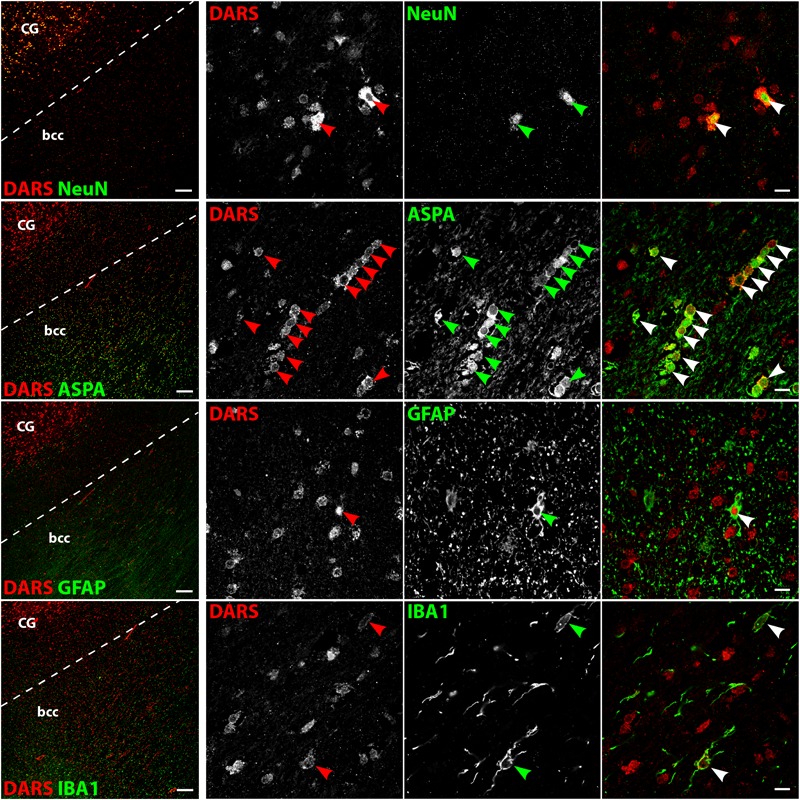
Cell type-specific DARS expression in the corpus callosum. Immunofluorescence of DARS (red) and the cell type-specific markers (green) NeuN (neurons), ASPA (oligodendrocytes), GFAP (astrocytes), and IBA1 (microglia). Left panel shows low power magnification of the body of corpus callosum (bcc) and the cingulate gyrus (CG; scale bar: 200 μm). Right panel displays high power images including separate channels to illustrate cell type-specific as well as subcellular DARS localization (scale bar: 10 μm). Arrowheads indicate DARS-positive cells.

The white matter of the body of the corpus callosum (bcc) showed little DARS immunoreactivity compared to the gray matter of the neighboring CG (**Figure 7**, left column). A common feature of white matter is the high abundance of myelinated axons with very few neuronal cell bodies. The neuronal somata that can be found in the bcc, however, were strongly DARS positive (**Figure 7**, top row). The vast majority of cells of the corpus callosum are oligodendrocytes. They are often located in strings of multiple cells and showed DARS expression in their thin cytoplasm (**Figure 7**, second row). The astrocytes (**Figure 7**, third row) and microglia (**Figure 7**, bottom row) present in the corpus callosum also revealed some weak DARS immunoreactivity.

### DARS Expression in the Striatum

**FIGURE 8:**
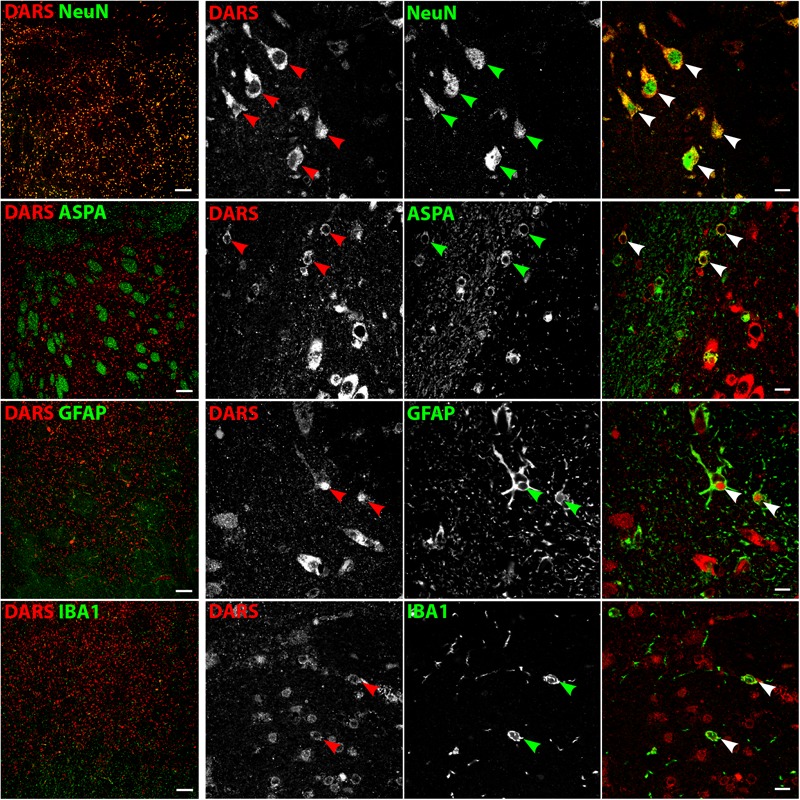
Cell type-specific DARS expression in the striatum. Immunofluorescence of DARS (red) and the cell type-specific markers (green) NeuN (neurons), ASPA (oligodendrocytes), GFAP (astrocytes), and IBA1 (microglia). Left panel shows low power magnification of the gray and white matter in the striatum (scale bar: 200 μm). Right panel displays high power images including separate channels to illustrate cell type-specific as well as subcellular DARS localization (scale bar: 10 μm). Arrowheads indicate DARS-positive cells.

Low power images of the striatum indicate a clear separation of DARS expression between the white and the gray matter (**Figure 8**, left column). While neurons of the gray matter showed a strong DARS signal in their cytoplasm (**Figure 8**, top row), limited DARS immunoreactivity was detected in the white matter bundles. High power images (**Figure 8**, right 3 columns) revealed that oligodendrocytes (**Figure 8**, second row), astrocytes (**Figure 8**, third row), and microglia (**Figure 8**, bottom row) express DARS, albeit at much lower levels compared to neurons. Interestingly, in some astrocytes of the striatum as well as other brain regions, DARS expression is not restricted to the cytoplasm but also seems to be present in the nucleus. To confirm a nuclear localization of DARS in these cells we performed a triple labeling of striatal astrocytes with DARS, GFAP, and the nuclear marker DAPI (**Supplementary Figure S1**) and found that in some astrocytes DARS was indeed located in the nucleus.

### DARS Expression in Human Stem Cell Derived Neural Cells

**FIGURE 9:**
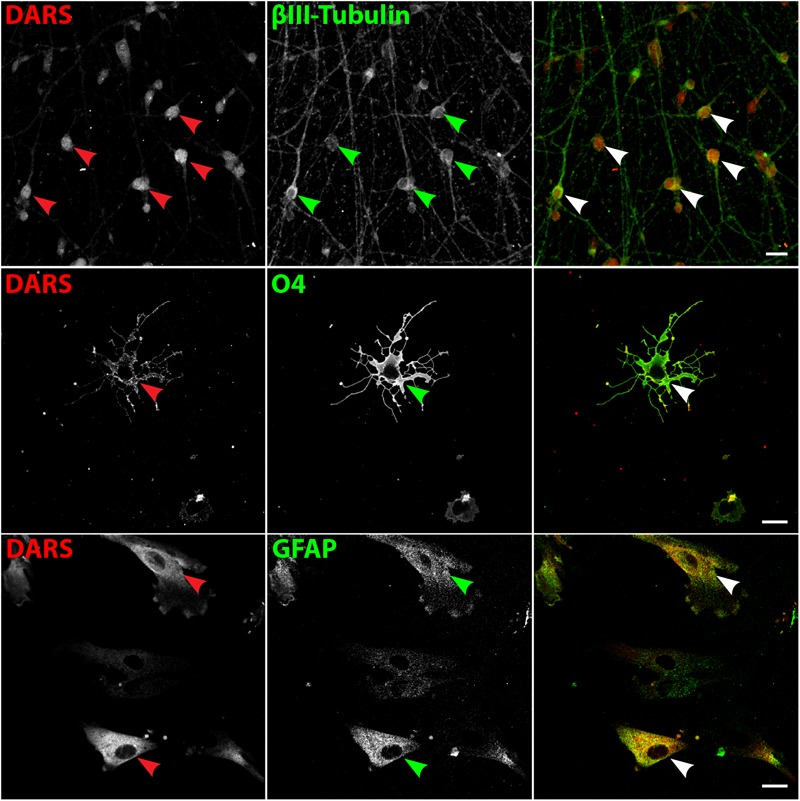
DARS expression in human stem cell-derived neural cells. Neurons differentiated from embryonic stem cells (hESCs) (Shi et al., 2012), oligodendrocytes differentiated from induced pluripotent stem cells (iPSCs) (Douvaras and Fossati, 2015), and astrocytes isolated from the human cerebral cortex (ScienCell Research Laboratories) were co-labeled for DARS (red) and the cell type-specific markers (green) βIII-Tubulin (neurons), O4 (oligodendrocytes), and GFAP (astrocytes; scale bar: 20 μm). Arrowheads highlight cells positive for DARS and the specific cell type marker.

To validate DARS expression levels in the major neural cell types *in vitro* we analyzed neurons differentiated from hESCs (Shi et al., 2012), oligodendrocytes differentiated from human iPSCs (Douvaras and Fossati, 2015), and human astrocytes isolated from the human cerebral cortex (obtained from ScienCell Research Laboratories). Expression of DARS in hESC derived neurons (**Figure 9**, top row; marked by βIII tubulin) and cultured primary human astrocytes (**Figure 9**, bottom row; marked by GFAP) was considerably higher than in iPSC-derived oligodendrocytes (**Figure 9**, second row; marked by O4 labeling). The subcellular localization of DARS in each of the neural cell types was predominantly cytoplasmic, with little DARS detected in the neuronal processes. In oligodendrocytes, on the other hand, a proportion of DARS could be detected within the extended cell processes, also referred to as myelin-like membranes.

## Discussion

Little is known about the natural distribution of ARSs in human tissues, which is a prerequisite to elucidate the complex etiology of ARS-deficiencies. This study presents a detailed biochemical and immunohistological characterization of DARS mRNA and protein expression at the regional and cellular level in the human brain. Broadly, our findings reveal similar DARS expression patterns in the mouse and human brain (Fröhlich et al., 2016). DARS is evolutionarily highly conserved across species and the amino acid sequence shows 96% homology between the human and the mouse ortholog. Therefore, matching anatomical distributions and cellular localizations were expected. This clear overlap in DARS gene expression between mouse and man will be beneficial for the development of HBSL mouse models that are relevant for clinical translation of treatment strategies in the future.

The highest DARS mRNA and protein levels were present in the cerebellum, a region responsible for the control of motor function. Consistently, DARS mutations in HBSL patients result in motor deficits with progressive leg spasticity and cerebellar dysfunction (Taft et al., 2013) indicating that this brain region might be particularly susceptible to disturbances in protein synthesis.

Interestingly, on a cell population level we observed much higher DARS expression levels in regions with high abundance of neuronal cell bodies compared to white matter tracts (**Table 1**). Accordingly, on a single cell level DARS expression in the CNS is significantly higher in neurons compared to oligodendrocytes, astrocytes, and microglia, which is consistent with our previous findings in the mouse (Fröhlich et al., 2016). The variable expression levels observed in glial cells and neurons might reflect subtype-specific differences within the particular lineage. Mature oligodendrocytes, for example, can be subdivided into six major, genetically distinct groups potentially with specific functions similar to neuronal subclasses (Marques et al., 2016). Depending on their function and location in the brain, some subtypes might have a higher protein turnover and therefore a higher demand for components of the translational machinery including DARS.

A hallmark of HBSL is the severe hypomyelination of the supratentorial white matter, the cerebellar peduncles, and the brainstem (Taft et al., 2013). To understand the complex pathology of the disease, the underlying cause of hypomyelination will need to be resolved. The relatively low DARS expression levels observed in oligodendrocytes, paired with their obligation to produce a vast amount of protein during the myelination process, could make these cells particularly vulnerable to enzyme dysfunction. This suggests that mutations causing even a slight reduction in DARS function might result in oligodendrocyte dysfunction and hypomyelination. Neurons with their relatively robust DARS expression, on the other hand, might be able to compensate for a partial loss of enzyme function with extra copies of the protein as long as DARS function is not completely abolished. In the CNS, neurons and oligodendrocytes form an intimate connection enabling bidirectional communication and support between the two cell types (Saab and Nave, 2017). It has been shown before that neuronal activity regulates oligodendrocyte differentiation as well as myelination (Gibson et al., 2014). Therefore, a primary DARS dysfunction in neurons, the cells with the highest expression levels, might lead to a secondary disturbance of oligodendrocyte function and ultimately demyelination as observed in HBSL. Most of the HBSL-causing mutations identified to date are located within the catalytic domain of the enzyme and could therefore either lead to a general disruption of protein synthesis or to mischarging of aspartate with incorrect tRNAs resulting in an accumulation of non-functional, unfolded, and misfolded proteins within the endoplasmic reticulum. The accumulation of this “protein junk” might overload the cellular degradation systems as well as the adaptive unfolded protein response, which then will trigger apoptosis to eliminate the malfunctioning cells (Lin and Popko, 2009).

The predominant subcellular localization of DARS in all neural cells investigated here appears to be cytoplasmic. In some astrocytes, however, a proportion of DARS also localized to the nucleus. The presence of ARSs in the nucleus has been described before (Kisselev and Wolfson, 1994) and it has been shown that tRNA charging is not restricted to cytoplasm and mitochondria but can also occur in the nucleus where it might provide a mechanism of “functional proofreading” of newly synthesized tRNAs before their export from the nucleus (Lund and Dahlberg, 1998). Alternatively, the occurrence of DARS in the nucleus of astrocytes might indicate a secondary, non-canonical function in this cell-type. This possibility is supported by a recent report on tryptophan-tRNA synthetase WARS mediating interferon-γ induced activation of the p53 signaling pathway in the nucleus (Sajish et al., 2012). It is possible that other ARSs including DARS exhibit similar secondary functions and influence major signaling pathways inside and outside the nucleus. In human stem cell derived oligodendrocytes a proportion of DARS was localized in the extended cell processes, which suggests a potential requirement for local protein translation upon myelination of axons. In agreement with this, recent transcriptome analysis of mouse CNS myelin has unraveled the presence of mRNAs coding for DARS, others ARSs, AIMP1, and components of the translation machinery within the myelin compartment along with mRNAs of the major myelin proteins (Thakurela et al., 2016).

This pioneering study of DARS expression domains in the human brain offers support to the idea of establishing HBSL animal models as well as testing targeted therapeutic interventions in these models. We have shown before that the complete knockout of DARS in mice is embryonically lethal and therefore not informative as a model for HBSL (Fröhlich et al., 2016). An improved strategy will be the introduction of HBSL-specific point mutations into the mouse *Dars* gene. The inter-species sequence homology and coinciding expression domains provide validatory support for this approach. The fact that HBSL is caused by single gene mutations makes it an ideal candidate for gene therapy. It has been shown previously that employing adeno-associated viruses equipped with cell type-specific promoters can drive transgene expression specifically in all major neural cell types (von Jonquieres et al., 2013, 2016). These tools combined with the knowledge of the specific DARS expression pattern in the brain will be helpful for creating a cell type and brain region specific gene therapy for HBSL. This, in turn, might be applied to treat other ARS-deficiencies.

## Author Contributions

DF and MK designed the study and wrote the paper. DF, AS, CV, RH, and GvJ performed the research. DF, AS, CV, EW, CS, TF, GH, and MK analyzed the data. All authors read and approved the final manuscript.

## Conflict of Interest Statement

The authors declare that the research was conducted in the absence of any commercial or financial relationships that could be construed as a potential conflict of interest.

## Supplementary Material

FIGURE S1 Nuclear localization of DARS in a subset of astrocytes. Immunofluorescence of DARS (red), the astrocyte marker GFAP (green), and the nuclear marker DAPI (blue) in coronal sections of the striatum (scale bar: 10 μm). Arrowheads indicate DARS expression in the nucleus of astrocytes.

## References

[B1] Antonellis A. Green E. D. (2008). The role of aminoacyl-tRNA synthetases in genetic diseases. *Annu. Rev. Genomics Hum. Genet.* 9 87–107. 10.1146/annurev.genom.9.081307.164204 18767960

[B2] Berthonneau E. Mirande M. (2000). A gene fusion event in the evolution of aminoacyl-tRNA synthetases. *FEBS Lett.* 470 300–304. 10745085 10.1016/s0014-5793(00)01343-0

[B3] Dogan S. A. Pujol C. Maiti P. Kukat A. Wang S. Hermans S. (2014). Tissue-specific loss of DARS2 activates stress responses independently of respiratory chain deficiency in the heart. *Cell Metab.* 19 458–469. 10.1016/j.cmet.2014.02.004 24606902

[B4] Douvaras P. Fossati V. (2015). Generation and isolation of oligodendrocyte progenitor cells from human pluripotent stem cells. *Nat. Protoc.* 10 1143–1154. 10.1038/nprot.2015.075 26134954

[B5] Fröhlich D. Suchowerska A. K. Spencer Z. H. von Jonquieres G. Klugmann C. B. Bongers A. (2016). In vivo characterization of the aspartyl-tRNA synthetase DARS: homing in on the leukodystrophy HBSL. *Neurobiol. Dis.* 97(Pt A) 24–35. 10.1016/j.nbd.2016.10.008 27816769

[B6] Gibson E. M. Purger D. Mount C. W. Goldstein A. K. Lin G. L. Wood L. S. (2014). Neuronal activity promotes oligodendrogenesis and adaptive myelination in the mammalian brain. *Science* 344:1252304. 10.1126/science.1252304 24727982 PMC4096908

[B7] Guo M. Schimmel P. (2013). Essential nontranslational functions of tRNA synthetases. *Nat. Chem. Biol.* 9 145–153. 10.1038/nchembio.1158 23416400 PMC3773598

[B8] Kisselev L. L. Wolfson A. D. (1994). Aminoacyl-tRNA synthetases from higher eukaryotes. *Prog. Nucleic Acid Res. Mol. Biol.* 48 83–142. 7938555 10.1016/s0079-6603(08)60854-5

[B9] Lin W. Popko B. (2009). Endoplasmic reticulum stress in disorders of myelinating cells. *Nat. Neurosci.* 12 379–385. 10.1038/nn.2273 19287390 PMC2697061

[B10] Lund E. Dahlberg J. E. (1998). Proofreading and aminoacylation of tRNAs before export from the nucleus. *Science* 282 2082–2085. 9851929 10.1126/science.282.5396.2082

[B11] Marques S. Zeisel A. Codeluppi S. van Bruggen D. Mendanha Falcao A. Xiao L. (2016). Oligodendrocyte heterogeneity in the mouse juvenile and adult central nervous system. *Science* 352 1326–1329. 10.1126/science.aaf6463 27284195 PMC5221728

[B12] Mersmann N. Tkachev D. Jelinek R. Roth P. T. Mobius W. Ruhwedel T. (2011). Aspartoacylase-lacZ knockin mice: an engineered model of Canavan disease. *PLoS One* 6:e20336. 10.1371/journal.pone.0020336 21625469 PMC3098885

[B13] Saab A. S. Nave K. A. (2017). Myelin dynamics: protecting and shaping neuronal functions. *Curr. Opin. Neurobiol.* 47 104–112. 10.1016/j.conb.2017.09.013 29065345

[B14] Sajish M. Zhou Q. Kishi S. Valdez D. M. Jr. Kapoor M. Guo M. (2012). Trp-tRNA synthetase bridges DNA-PKcs to PARP-1 to link IFN-gamma and p53 signaling. *Nat. Chem. Biol.* 8 547–554. 10.1038/nchembio.937 22504299 PMC3780985

[B15] Scheper G. C. van der Klok T. van Andel R. J. van Berkel C. G. Sissler M. Smet J. (2007). Mitochondrial aspartyl-tRNA synthetase deficiency causes leukoencephalopathy with brain stem and spinal cord involvement and lactate elevation. *Nat. Genet.* 39 534–539. 10.1038/ng2013 17384640

[B16] Shi Y. Kirwan P. Livesey F. J. (2012). Directed differentiation of human pluripotent stem cells to cerebral cortex neurons and neural networks. *Nat. Protoc.* 7 1836–1846. 10.1038/nprot.2012.116 22976355

[B17] Taft R. J. Vanderver A. Leventer R. J. Damiani S. A. Simons C. Grimmond S. M. (2013). Mutations in DARS cause hypomyelination with brain stem and spinal cord involvement and leg spasticity. *Am. J. Hum. Genet.* 92 774–780. 10.1016/j.ajhg.2013.04.006 23643384 PMC3644624

[B18] Thakurela S. Garding A. Jung R. B. Muller C. Goebbels S. White R. (2016). The transcriptome of mouse central nervous system myelin. *Sci. Rep.* 6:25828. 10.1038/srep25828 27173133 PMC4865983

[B19] von Jonquieres G. Fröhlich D. Klugmann C. B. Wen X. Harasta A. E. Ramkumar R. (2016). Recombinant human myelin-associated glycoprotein promoter drives selective AAV-mediated transgene expression in oligodendrocytes. *Front. Mol. Neurosci.* 9:13. 10.3389/fnmol.2016.00013 26941604 PMC4763065

[B20] von Jonquieres G. Mersmann N. Klugmann C. B. Harasta A. E. Lutz B. Teahan O. (2013). Glial promoter selectivity following AAV-delivery to the immature brain. *PLoS One* 8:e65646. 10.1371/journal.pone.0065646 23799030 PMC3683058

[B21] Wolf N. I. Toro C. Kister I. Latif K. A. Leventer R. Pizzino A. (2015). DARS-associated leukoencephalopathy can mimic a steroid-responsive neuroinflammatory disorder. *Neurology* 84 226–230. 10.1212/WNL.0000000000001157 25527264 PMC4335995

[B22] Yao P. Fox P. L. (2013). Aminoacyl-tRNA synthetases in medicine and disease. *EMBO Mol. Med.* 5 332–343. 10.1002/emmm.201100626 23427196 PMC3598075

