# Supplementary figures and images for: Expression Pattern of the Aspartyl-tRNA Synthetase DARS in the Human Brain
Source: Front Mol Neurosci. 2018 Mar 20;11:81. doi: 10.3389/fnmol.2018.00081 (PMC5869200; doi:10.3389/fnmol.2018.00081)

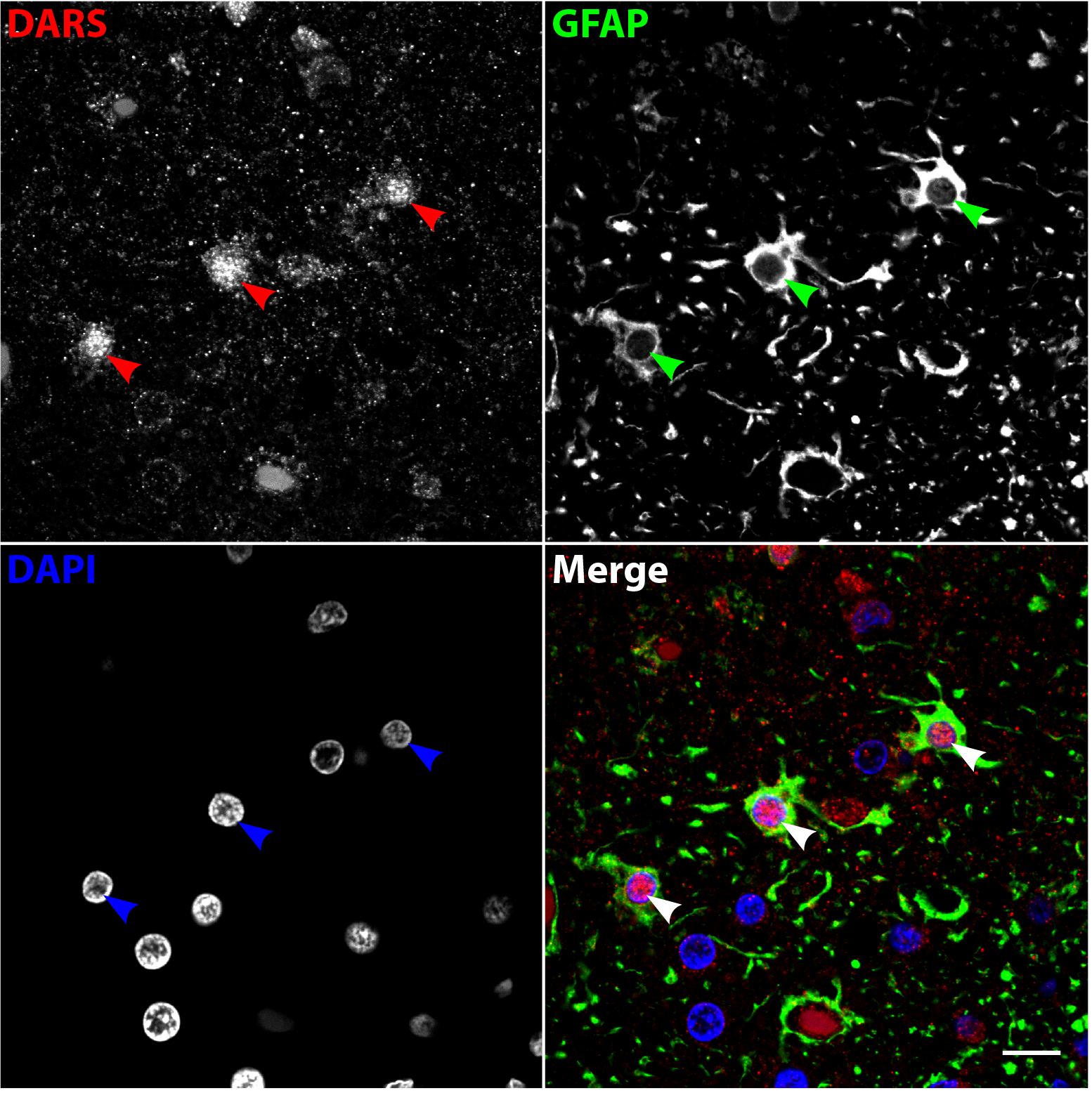

Supplement: FIGURE S1 — Nuclear localization of DARS in a subset of astrocytes. Immunofluorescence of DARS (red), the astrocyte marker GFAP (green), and the nuclear marker DAPI (blue) in coronal sections of the striatum (scale bar: 10 μm). Arrowheads indicate DARS expression in the nucleus of astrocytes. [file Image_1.TIF]
